# Molecular mechanisms of Sar/Arf GTPases in vesicular trafficking in yeast and plants

**DOI:** 10.3389/fpls.2014.00411

**Published:** 2014-08-21

**Authors:** Tomohiro Yorimitsu, Ken Sato, Masaki Takeuchi

**Affiliations:** ^1^Department of Life Sciences, Graduate School of Arts and Sciences, University of TokyoTokyo, Japan; ^2^Department of Chemistry, Graduate School of Science, University of TokyoTokyo, Japan

**Keywords:** small GTPase, vesicular trafficking, endoplasmic reticulum, Golgi apparatus, endosome

## Abstract

Small GTPase proteins play essential roles in the regulation of vesicular trafficking systems in eukaryotic cells. Two types of small GTPases, secretion-associated Ras-related protein (Sar) and ADP-ribosylation factor (Arf), act in the biogenesis of transport vesicles. Sar/Arf GTPases function as molecular switches by cycling between active, GTP-bound and inactive, GDP-bound forms, catalyzed by guanine nucleotide exchange factors and GTPase-activating proteins, respectively. Activated Sar/Arf GTPases undergo a conformational change, exposing the N-terminal amphipathic α-helix for insertion into membranes. The process triggers the recruitment and assembly of coat proteins to the membranes, followed by coated vesicle formation and scission. In higher plants, Sar/Arf GTPases also play pivotal roles in maintaining the dynamic identity of organelles in the secretory pathway. Sar1 protein strictly controls anterograde transport from the endoplasmic reticulum (ER) through the recruitment of plant COPII coat components onto membranes. COPII vesicle transport is responsible for the organization of highly conserved polygonal ER networks. In contrast, Arf proteins contribute to the regulation of multiple trafficking routes, including transport through the Golgi complex and endocytic transport. These transport systems have diversified in the plant kingdom independently and exhibit several plant-specific features with respect to Golgi organization, endocytic cycling, cell polarity and cytokinesis. The functional diversification of vesicular trafficking systems ensures the multicellular development of higher plants. This review focuses on the current knowledge of Sar/Arf GTPases, highlighting the molecular details of GTPase regulation in vesicle formation in yeast and advances in knowledge of the characteristics of vesicle trafficking in plants.

## Introduction

Eukaryotes utilize guanine nucleotides to regulate many intracellular cellular processes, including the endomembrane vesicular trafficking system. In general, trafficking is turned on when a “switch” molecule is activated by binding to GTP; in contrast, the system is off when the molecule is in the inactive, GDP-bound form. This on-off action is cycled consecutively via control of the activity of the switch molecule. To maintain homeostasis, cells require the integrity of membrane trafficking resulting from accurate switch activity. Secretion-associated Ras-related (Sar) and ADP-ribosylation factor (Arf) of small GTPase family proteins belong to the Ras superfamily and serve as such switch molecules for the precise operation of vesicular trafficking systems. The Sar/Arf proteins are highly conserved among species from yeast to mammals as well as in plants and are classified based on amino acid sequence homology. Sar1 was first identified as a multicopy suppressor of a temperature-sensitive *sec12* mutant in *S. cerevisiae* (Nakano et al., 1988). Higher eukaryotes have more than two Sar1 orthologs (e.g., two genes in vertebrates), whereas *S. cerevisiae* has only one Sar1. In contrast, several Arf genes are found in various species (three genes in yeast and six in mammals). The first Arf, Arf1, was cloned from bovine and yeast and has been identified as the cofactor for activating the ADP-ribosylation of a heteromeric G protein by cholera toxin *in vitro* (Kahn et al., 1988; Sewell and Kahn, 1988). Arf proteins are categorized into three classes. Although mammals have all classes, Classes I, II and III, yeast and plants lack Class II. Arf1, belonging to Class I, is the best studied, especially with regard to its role in vesicular trafficking.

To date, a large number of genes of the Sar/Arf proteins have been identified in plants (Jurgens and Geldner, 2002; Vernoud et al., 2003), and the complementation of yeast mutants has been a useful tool to isolate and characterize these genes (D'Enfert et al., 1992; Kim et al., 1997; Takeuchi et al., 1998, 2000, 2002; De Craene et al., 2014). Regulators and other interacting proteins of these GTPases have also been identified in plants by the use of yeast mutants and amino acid sequence similarity to yeast and mammalian orthologs (Vernoud et al., 2003). In the genome of the model plant *Arabidopsis thaliana*, four *SAR1* genes exist, which form a small gene family; in contrast, there are 12 *ARF* genes, comprising a multiple gene family (Robinson et al., 2007). In comparison with *Arabidopsis* Sar1 proteins, *Arabidopsis* Arf proteins appear to be involved in many different vesicular trafficking routes. Their trafficking systems have diversified in the plant kingdom independently of other organisms and are deeply involved in several plant-specific features. Thus, the functional diversification of vesicular trafficking systems is key to understanding the multicellular development of higher plants.

### Sar1/Arf1 small GTPases

Sar1/Arf1 proteins bidirectionally manage vesicular trafficking in the early secretory pathway between the ER and Golgi: the anterograde pathway from the ER to Golgi depends on Sar1, whereas the opposite retrograde pathway from the Golgi to ER depends on Arf1. Distinct sets of vesicles move forward and backward and transfer proteins and lipids via these pathways. The transport vesicles are covered with distinct sets of coat protein (COP) complexes and bud specifically from donor organelle membranes (Brandizzi and Barlowe, 2013). COPII-coated vesicles are derived from the ER membrane and carry substances to the Golgi, and COPI-coated vesicles are derived from Golgi membranes to mediate transport to the ER. To ensure proper vesicle formation, COPI and COPII proteins consisting of completely different components should be properly recruited to each organelle membrane. For this purpose, Sar1/Arf1 proteins are switched on and off as appropriate on the respective organelle membranes by specific regulators that convert their guanine nucleotide-binding state.

**Figure 1:**
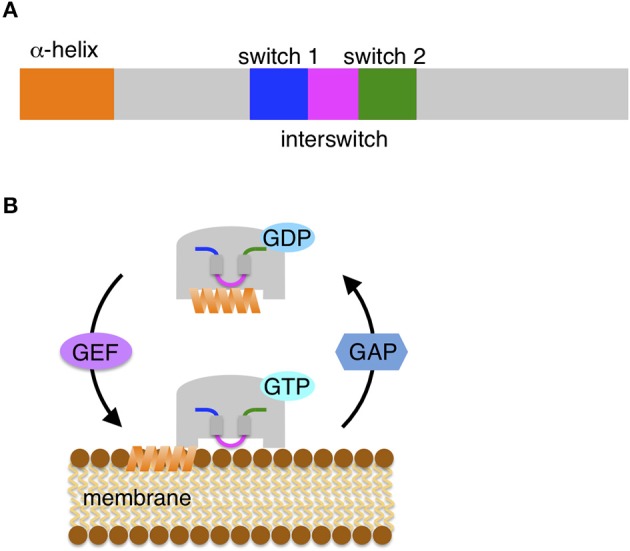
**Small GTPase Sar1/Arf1 protein. (A)** Schematic diagrams of Sar/Arf. Conserved domains are depicted: the N-terminal amphipathic α-helix, two switch regions (switch 1 and switch 2) and the interswitch region. **(B)** The Sar/Arf protein cycles between membrane-association and dissociation. GDP-bound cytosolic Sar/Arf is inactive and carries the N-terminal amphipathic helix in a hydrophobic pocket. A guanine nucleotide exchange factor (GEF) mediates the exchange of GDP for GTP in Sar/Arf. GTP-loaded Sar/Arf undergoes a conformational change of the two switch and interswitch regions, triggering the extrusion of the helix from the pocket. Subsequently, the shallow insertion of the amphipathic helix into the outer leaflet of the lipid bilayers allows Sar/Arf to associate tightly with the membrane surface. For dissociation, GTPase activating protein (GAP) activates the GTP hydrolysis activity of Sar/Arf. Conserved domains are shown in the same color in **(A)** and **(B)**.

Sar1/Arf1 proteins are primarily responsible for the recruitment of COP proteins to membranes and initiation of the formation of COP-mediated vesicles. These proteins share structural similarity despite their different sequence identity (Figure 1A). At the initial step of COP vesicle formation, the two proteins function as switch molecules by similar mechanisms. The Sar1/Arf1 proteins have a characteristic α-helix at the N-terminal end that is composed of approximately 20 hydrophobic and hydrophilic amino acid residues, resulting in its amphipathic nature (Antonny et al., 1997; Bielli et al., 2005; Lee et al., 2005). When bound to GDP, Sar1/Arf1 proteins are cytosolic and inactive: the amphipathic α-helix is sequestered in a hydrophobic pocket on the surface. The exchange of GDP for GTP induces a conformational change into the active form (Figure 1B); in this conformation, a loop region flanked by β-sheets between two switch domains (the so-called interswitch region) is displaced from the nucleotide-binding site, which forces exposure of the helix out of the hydrophobic pocket (Goldberg, 1998; Bi et al., 2002). However, the extruded helix requires engagement in a suitable hydrophobic environment because of its amphipathicity. As a consequence of the hydrophobic face of the helix being laterally inserted into the outer leaflet of the lipid bilayer, GTP-loaded active Sar1/Arf1 proteins associate stably with the donor organelle membrane.

The N-terminal helix is also used to deform the membrane (Bielli et al., 2005; Lee et al., 2005; Beck et al., 2008; Krauss et al., 2008; Lundmark et al., 2008). *In vitro* experiments show that when mixed with purified Sar1/Arf1 protein, liposomes are deformed into a highly curved tubular structure. This tubulation process requires the hydrophobicity of the N-terminal amphipathic helix. In contrast, a Sar1 mutant lacking the N-terminal helix is still able to deform an artificial liposome membrane *in vitro* (Stachowiak et al., 2012). When chemically bound to the lipid, the Sar1 mutant protein accumulates in the subdomain; in this case, the crowding of Sar1 on the membrane surface drives the tubulation. Tubular structures could be involved physiologically with non-vesicular carriers for ER-Golgi transport, as suggested by several observations in various organisms, including yeast (Fatal et al., 2004; Mironov, 2014). Further study is necessary for a better understanding of a role of Sar1/Arf1-formed tubular structures in biological processes *in vivo*.

**Figure 2:**
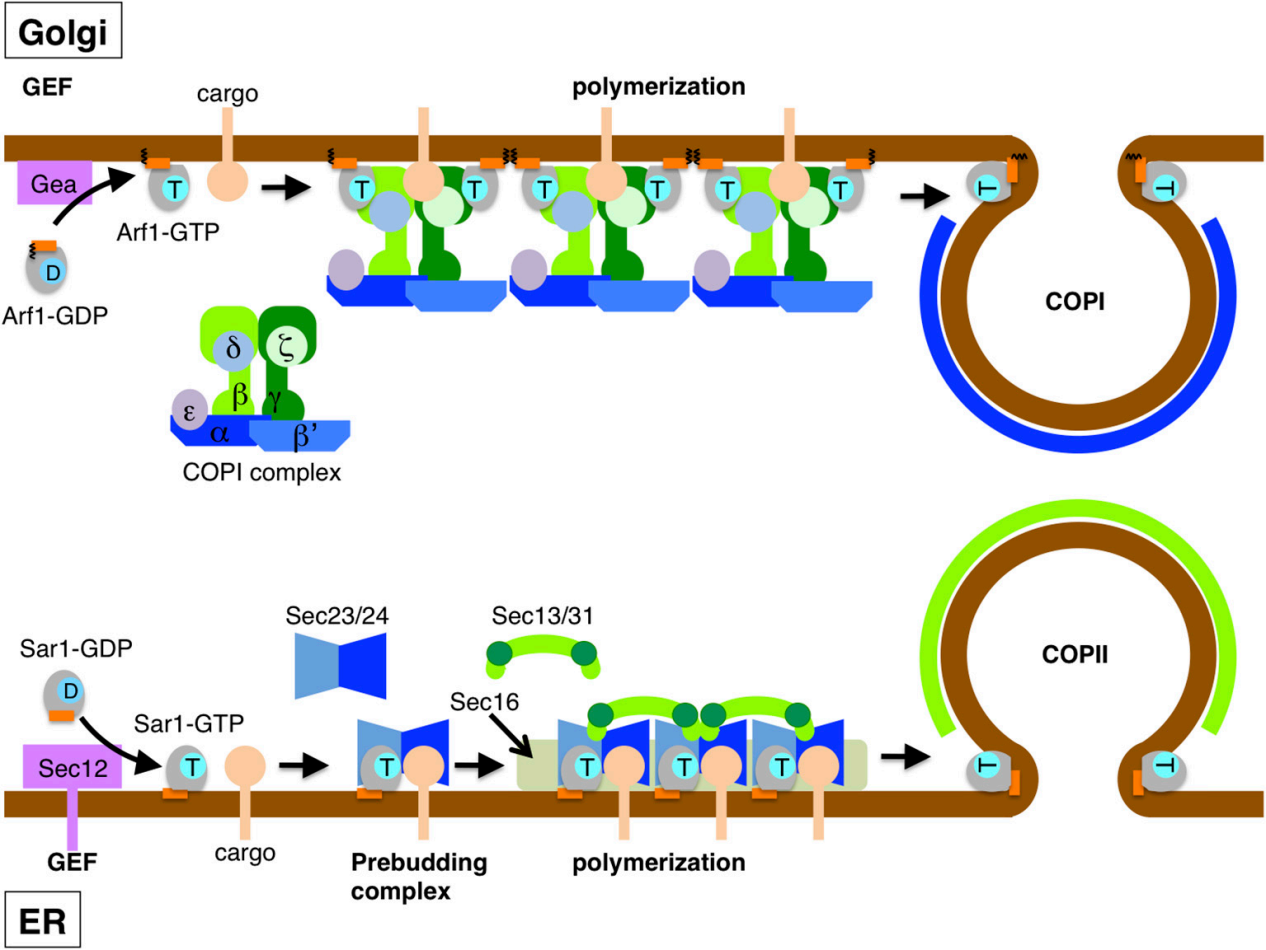
**Assembly of COPII and COPI coats drives vesicle formation**. Vesicle formation starts upon the recruitment of Sar1 and Arf1 to the ER (lower) and Golgi membranes (upper), respectively. In COPII vesicle formation, the ER integral membrane protein Sec12 exchanges GDP for GTP bound to Sar1 through its GEF activity. Membrane-associated GTP-bound Sar1 recruits the inner coat Sec23/24 complex and then assembles along with cargo protein into the pre-budding complex. Outer coat Sec13/31 complexes are recruited to the pre-budding complexes and self-assembled by crosslinking. The polymerization of Sec13/31 by self-assembly drives membrane curvature to form a spherically shaped vesicle. COPI vesicle formation is also initiated by GTP-GDP exchange on Arf1 through the action of the GEF Gea protein (Gea1 or Gea2), which is peripherally located on the Golgi membrane. GTP-bound Arf1 stably binds to the membrane by a myristoylated amphipathic helix, as does Sar1. The heptamer complex of the COPI coat is recruited *en bloc* and associates with cargo as well as two Arf1 molecules though the inner layer coat complex (β/γ/δ/ζ-COP). As in COPII, vesicles are formed upon polymerization of the outer coat (α/β′/ε-COP). The amphipathic helix of Sar1 and Arf1 has some role in the scission of budded vesicles.

Arf family proteins such as Arf1 possess a modification of a myristoyl moiety on the N-terminal helix, which is required for potential biological activity as well as membrane association (Kahn et al., 1988, 1995; Franco et al., 1996) (Figure 2). Sar1 does not undergo such modifications. Myristoylation occurs cotranslationally on the N-terminal second glycine residue of Arf exposed after cleavage of the first methionine residue, which contributes to increased hydrophobicity of the N-terminal amphipathic helix. Without the exposed helix, GDP-loaded inactive Arf1 associates unstably with membranes solely through the myristoyl group. Hence, the hydrophobic nature due to both is required for stable membrane association of GTP-bound Arf1.

### Guanine nucleotide exchange factors

In the presence of Mg^2+^ and liposomes, the spontaneous exchange of GDP to GTP occurs efficiently in Sar1/Arf1 proteins *in vitro* (Barlowe et al., 1993; Franco et al., 1996). However, GDP-GTP exchange *in vivo* relies on a catalytically assisting protein called guanine nucleotide exchange factor (GEF). Because free GTP is much more abundant than free GDP in cells, the release of GDP from a nucleotide-binding site is sufficient to complete GDP-GTP exchange. A specific GEF protein catalyzes the conversion from the inactive state to active state of Sar1 and Arf1 on the appropriate organelle membrane (Figure 1B). Sec12 is a type II transmembrane protein localizing at the ER and exclusively acts as the GEF for Sar1. Sec12 was originally identified from yeast mutants defective in ER-Golgi transport (Novick et al., 1980). Its catalytic domain facing the cytosol is composed of a seven-bladed β-barrel structure, from which a loop with K^+^ bound (termed the K loop) extends (McMahon et al., 2012). Catalytically essential residues have been identified around the K loop. Accordingly, it is proposed that Sec12 contacts GDP-loaded Sar1 through the loop, mediating the conversion of the GDP-bound to GTP-bound form of Sar1. The transmembrane domain of Sec12 has an important role in its appropriate ER localization, ensuring strict Sar1 recruitment to the ER membrane. In yeast, when Sec12 escapes from the ER, the Golgi protein Rer1 retrieves Sec12 back to the ER by recognizing its transmembrane domain (Sato et al., 1996).

In contrast, Arf GEF proteins are more diverse (Anders and Jurgens, 2008). Unlike Sec12, Arf GEF proteins localize peripherally to membranes and possess a conserved Sec7 domain to exert their GEF activity. In yeast, Arf1 has four GEF proteins, and two of them, Gea1 and Gea2, play a redundant role in Arf1 activation to regulate COPI vesicle formation (Peyroche et al., 1996, 2001). The Gea protein is soluble but is partially recruited to *cis*-Golgi membranes because of cycling between the cytosol and membrane. The Golgi transmembrane protein Gmh1 was identified as an interactor of Gea and a potential candidate for the membrane recruitment of the Gea protein (Chantalat et al., 2003). However, as Gmh1 depletion did not have a strong effect on the membrane association of the Gea protein, it remains unknown how Gea associates with membranes. Although *in vitro* reconstitution experiments have clearly demonstrated that Sec12 and Gea constitutively facilitate GDP-GTP exchange in Sar1 and Arf1, respectively (Peyroche et al., 1996; Futai et al., 2004), it is unclear whether and how such catalytic activity is controlled *in vivo*.

### GTPase activating proteins and their regulation

GTP-locked mutant forms of Sar1 and Arf1 display dominant-negative effects, indicating that accomplishment of the GTPase cycle is physiologically essential (Kahn et al., 1995; Saito et al., 1998). The Sar1/Arf1 protein displays little or no intrinsic GTP hydrolysis activity, though each protein has specific GTPase-activating protein (GAP) partners, which simply evoke a reaction opposite to that of GEF (Figure 1B). When a GAP activates the GTP hydrolysis activity, the Sar1/Arf1 protein is inactivated and dissociates from the membrane. However, their physiological functions extend beyond that.

After association with the ER membrane, GTP-bound active Sar1 recruits the COPII coat subunit of the Sec23/24 heterodimer complex from the cytosol (Matsuoka et al., 1998) (Figure 2). Of the complex, Sec23 forms a direct interaction with Sar1 and also acts as its GAP (Yoshihisa et al., 1993). The crystal structure reveals the molecular mechanism by which Sec23 stimulates Sar1 GTPase activity, whereby Sec23 inserts a key arginine residue into the active site of Sar1 (Bi et al., 2002). However, the Sec23-stimulated GTPase activation of Sar1 is relatively inefficient for triggering full coat disassembly (Antonny et al., 2001). Sec24 captures the transmembrane cargo protein and the adaptor/receptor protein for the soluble cargo existing in the ER lumen by binding to the cytosolic tail (Miller et al., 2003; Mossessova et al., 2003). The Sar1/Sec23/24/cargo complex, termed the pre-budding complex, is relatively stable enough to prevent coat disassembly. Conversely, when the Sar1/Sec23/24 complex fails to capture cargo by Sec24 dissociating from the membrane, each dissociated protein is recycled again to form the pre-budding complex properly (Koizumi et al., 2005). In yeast, an ER integral membrane protein, Sed4, potentially plays some role in this process (Espenshade et al., 1995; Kodera et al., 2011). Sed4 interacts directly with Sar1 but has no GEF activity for Sar1, despite high similarity of the N-terminal, cytosolic domain with Sec12. Instead, Sed4 exhibits stimulation of Sec23-mediated as well as intrinsic Sar1 GTPase activity and the acceleration of coat disassembly only in the absence of cargo proteins. Thus, Sed4 is proposed to have a role in efficient recycling of the coat and Sar1 by disassembly of the Sar1-Sec23/24 complex that is free of cargo. Further study is required to clarify the mechanisms of Sar1 GTPase activation by Sed4.

At the next step, the prebudding complex recruits Sec13/31 heterotetramer complexes, which form the outer coat, to cross-link the adjacent prebudding complexes via polymerization (Matsuoka et al., 1998; Tabata et al., 2009) (Figure 2). A cryo-electron microscopy study has revealed that purified Sec13/31 complexes self-assemble to form a spherical lattice-like structure in solution, the size and shape of which closely fit with those of COPII vesicles (Stagg et al., 2006). Accordingly, lateral Sec13/31 polymerization could incorporate the cargo into a nascent vesicle and simultaneously drive membrane curvature to form the precise vesicular shape. In addition to the scaffolding role, Sec31 acts as the Sec23 GAP stimulator. The crystal structure of the active fragment of Sec31 with the Sar1/Sec23 complex reveals insight into the mechanisms of GAP stimulation (Bi et al., 2007). Sec31 has a C-terminal proline-rich domain as the GAP stimulator. Within the complex, this domain binds across the extended surface of Sec23 and Sar1 and accesses the active site to optimize the orientation of the catalytically important histidine residue of Sar1. Taken together, there are two-step processes for Sec23 GAP activity and Sec31 GAP stimulation for the full activation of Sar1 GTP hydrolysis.

This two-step activation system has been successfully reproduced in minimal reconstitution experiments with liposomes (Antonny et al., 2001). In these experiments, however, full activation of the Sar1 GTPase causes the membrane-associated coat to be rapidly disassembled. To overcome this paradoxical situation, there are at least two potential solutions, as mentioned above: the constant activation of Sec12 GEF and a contribution by cargo molecules to stabilizing the prebudding complex (Futai et al., 2004; Koizumi et al., 2005). In addition, a peripheral ER-membrane protein, Sec16, acts as a GAP inhibitor to contribute to stable coat assembly. Sec16 is an essential protein for COPII vesicle formation *in vivo* and interacts with all of the COPII coat proteins through its multiple domains at vesicle-formation sites on the ER and ER exit sites (Espenshade et al., 1995; Shaywitz et al., 1997; Supek et al., 2002; Connerly et al., 2005). Recent studies have reported that Sec16 not only functions as a recruiter of the coat but also modulates the interaction of Sec31 with the Sar1/Sec23/24 complex (Kung et al., 2012; Yorimitsu and Sato, 2012). Although the detailed mechanisms still remain elucidated, Sec16 might interfere with the catalytic interaction between the active domain of Sec31 and the Sar1/Sec23 complex.

GTP-bound Arf1 primes COPI coat assembly to the Golgi membrane. The COPI coat is composed of seven proteins, α- (Cop1), β- (Sec26), β′- (Sec27), γ- (Sec21), δ- (Ret2), ε- and ζ-COP (Ret3), with the corresponding yeast proteins in parentheses. Although biochemically separable into two subcomplexes of α/β′/ε-COP and β/γ/δ/ζ-COP, the COPI coat complex is recruited *en bloc* to the Golgi membrane through the direct interaction with membrane-bound Arf1 (Hara-Kuge et al., 1994) (Figure 2). α/β′/ε-COP forms the cage-like structure of the outer layer coat that resembles Sec13/31 and clathrin structures (Lee and Goldberg, 2010). β/γ/δ/ζ-COP serves as the inner coat to capture cargo proteins, with γ/ζ-COP being structurally similar to the α/σ adaptins of the AP2 clathrin adaptor (Yu et al., 2012). Two molecules of Arf1 interact with the inner COPI coat through the β-COP and γ-COP subunits. Although likely analogous to the COPII coat, COPI itself has no GAP function. Alternatively, the GAP protein ArfGAP1 separately serves as the Arf1 GTPase activator in COPI vesicle formation. Similar to the COPII systems, the β-COP and γ-COP coat subunits possess the ability to promote the GAP activity of Arf1 in solution (Yu et al., 2012), although the mechanisms remain to be elucidated. In reconstitution systems with synthetic liposomes, GAP is not always essential for vesicle formation (Spang et al., 1998). In other cases, GAP promotes COPI vesicle formation, with cargo proteins having a key role. Similar to the step of the pre-budding complex formation of COPII, in a situation in which there is no cargo, Arf1 GTP hydrolysis drives the dissociation of the coat from the membrane and then reuses it until cargo is captured.

Successful Arf1/COPI/cargo complexes move to the vesicle-forming step by coat polymerization, and finally cargo-incorporated COPI vesicles bud and form (Spang et al., 2010; Shiba and Randazzo, 2012). Gcs1 and Glo3 in yeast serve as the Arf1 GAP in COPI vesicle formation. These two proteins are distinct in structure and partially overlap in function (Poon et al., 1999). Gcs1 belongs to the ArfGAP1 family, whereas Glo3 belongs to the ArfGAP2/3 family; both have conserved GAP catalytic domains at the N-terminus. Only Gcs1 has a specific lipid-binding motif at the C-terminus, which mediates the preferential association with highly curved membranes, which is suggested to have some role in regulating Gcs1 GAP activity (Bigay et al., 2005). In contrast, Glo3 does not have an obvious lipid-binding motif. Yeast mutants lacking either the *GCS1* or *GLO3* gene grow well, whereas the double-deletion mutant is lethal (Poon et al., 1999). Gcs1 and Glo3 suppress the lethality of an Arf1-malfunctional mutant when overexpressed and may also have a potential function in the formation of the Arf1/COPI coat/cargo complex, which again supports the idea that Arf1 GAP plays a positive role in vesicle formation (Zhang et al., 1998). The precise functions of GAP remain controversial, and further studies are required for a comprehensive model.

As mentioned above, the functional role of GTP hydrolysis is in the efficient formation of selective cargo-incorporated vesicles by cycling the disassembly and assembly of the coat to allow its successful capture of cargo. A second role is suggested in the scission of the budded vesicle from the membrane. It was shown that the release of the COPII vesicle is inhibited in the presence of a Sar1 mutant lacking the N-terminal amphipathic helix or GTP hydrolysis activity. However, there is conflicting evidence showing that vesicles are successfully formed in the presence of the non-hydrolyzable GTP analog GMP-PNP. Another role is coat dissociation from the completed vesicle prior to arrival at the acceptor organelle. Fusion with the organelle membrane requires at least a partially uncoated, naked part on the vesicle membrane. Uncoating due to the inactivation of Sar1/Arf1 proteins can employ activated GAP. However, it is unknown when and how GAP is activated to hydrolyze GTP on Sar1/Arf1 proteins. It was shown that only a small amount of Sar1 proteins are detected from isolated formed vesicles (Barlowe et al., 1994). It is possible that during budding and/or just before completion, at least partial portion of the Sar1/Arf1 proteins were released from the membrane surface of coated vesicles by the action of GAP. This fits with the observation that the TRAPPI complex and Ypt1, which serve in the tethering event, can bind to COPII vesicles through the interaction with Sec23 after Sar1 is depleted (Cai et al., 2007; Lord et al., 2011). Subsequently, at the Golgi surface, the Hrr25 protein kinase, in association with the Golgi, phosphorylates Sec23/24 to release the coat and eventually promote vesicle fusion. In this model, however, it remains unknown how coat proteins are retained on the forming and formed vesicles without the action of Sar1.

### Plant vesicular trafficking

In plant cells, the secretory pathway exports a variety of proteins to the cell surface and is essential for the expansion and elongation of the cells. The major molecular components in secretory systems are well conserved among eukaryotes. However, the morphological properties of the secretory organelles show great divergence between plants and mammals. The characteristic features of plant secretion, such as the formation of cell plates during cytokinesis, a polydisperse mobile Golgi apparatus and the lack of an intermediate compartment between the ER and the Golgi apparatus, expand many plant-specific molecules in the maintenance and regulation of vesicular trafficking through the secretory pathway. The development and morphogenesis of higher plants require the strict regulation of vesicular trafficking. To elucidate vesicular trafficking in plants, fluorescent proteins have been extensively utilized for the visualization of proteins localizing at various membrane-bound compartments. The advent of live cell imaging utilizing fluorescently tagged proteins has provided unprecedented insight into the movement of proteins and their interactions in plant studies.

### Plant Sar1 proteins and their interacting proteins

To investigate the roles of Sar1 proteins in plant cells, a system that utilizes their dominant-negative mutants was established. From the knowledge accumulated in yeast and mammalian studies, mutants that are virtually fixed at the GTP- or GDP-bound state, which act dominantly over the wild-type protein, were constructed. By the transient expression of such dominant mutants of *Arabidopsis* Sar1 protein (AtSar1) and green fluorescent protein (GFP)-tagged marker proteins, it was demonstrated that AtSar1 is required for transport from the ER to the Golgi apparatus (Takeuchi et al., 2000). This transient expression system in plant cells also provides a tool to manipulate membrane traffic by GTPase mutants, even when their effects are toxic for cell growth. Similar molecular approaches have also been successfully applied for the study of other GTPases and secretory genes (Batoko et al., 2000; Phillipson et al., 2001). Regardless, it took time before the conditional expression of dominant-negative plant Sar1 became possible in stable transgenic plants (Osterrieder et al., 2010). The inducible expression of a GTP-locked mutant of tobacco Sar1 enabled the investigation of protein dynamics after the blockade of ER-to-Golgi transport at the electron microscopic level (Osterrieder et al., 2010).

The *Arabidopsis* genome encodes several COPII components, four Sar1, seven Sec23, three Sec24, two Sec13, and two Sec31 (Robinson et al., 2007) (Table 1). Regarding Sar1, Sec24 and Sec13 isoforms, functional complementation was reported through yeast expression studies (D'Enfert et al., 1992; Takeuchi et al., 1998; De Craene et al., 2014). A functional heterogeneity among plant Sar1 isoforms was reported for the function of plant Sar1 (Hanton et al., 2008). Yellow fluorescent protein (YFP) fusions of two *Arabidopsis* Sar1 isoforms were differently localized at the ERES and showed different levels of partition with the membrane fraction. In addition, functional analyses using a secretion marker protein indicated that the overexpression of GTP-locked mutants of the two Sar1 isoforms caused different levels of ER export inhibition (Hanton et al., 2008). In rice (*Oryza sativa*), four Sar1 isoforms (OsSar1a, OsSar1b, OsSar1c, and OsSar1d) were cloned and characterized (Tian et al., 2013). Gene suppression experiments by RNA interference revealed that single knock down of one of the OsSar1 isoforms showed no obvious phenotype but simultaneous knock down of OsSar1 a/b/c resulted in floury and shrunken seeds and caused the generation of numerous novel protein bodies with highly electron-dense matrixes containing both glutelin and α-globulin in the endosperm of transgenic plants, suggesting the presence of a functional redundancy among rice Sar1 isoforms (Tian et al., 2013).

**Table 1:** ***Arabidopsis* Sar1 proteins and their interacting proteins**.

**Protein**	**Other name**	**AGI numbers**	**Function or putative function**
AtSARA1a	AtSar1	At1g09180	Small GTPase
AtSARA1b		At1g56330	Small GTPase
AtSARA1c	AtSar2	At4g02080	Small GTPase
AtSARA1d		At3g62560	Small GTPase
Sec12		At2g01470	Guanine nucleotide exchange factor (GEF) for Sar1
Sec23		At3g23660	GTPase-activating protein (GAP) for Sar1
		At1g05520	
		At5g43670	
		At4g14160	
		At2g21630	
		At4g01810	
		At2g27460	
Sec24		At3g07100	Coat protein for COPII vesicle
		At3g44340	
		At4g32640	
Sec13		At3g01340	Coat protein for COPII vesicle
		At2g30050	
Sec31		At1g18830	Coat protein for COPII vesicle
		At3g63460	
Sec16		At5g47480	Scaffold protein at ER exit sites
		At5g47490	

*Arabidopsis* plants possess three types of Sec24 isoforms (AtSec24A, AtSec24B and AtSec24C) (Robinson et al., 2007). An *Arabidopsis* missense recessive mutation in *sec24A* showed an aberrant phenotype, with partially accumulated Golgi membrane markers and a soluble secretory marker in globular structures composed of large amounts of convoluted ER membranes (Faso et al., 2009; Nakano et al., 2009). Only AtSec24A, but not other AtSec24 isoforms, could complement these mutant phenotypes. The complete loss of *sec24A* function led to a lethal phenotype, suggesting that *AtSEC24A* is an essential gene. In contrast, AtSec24B knockout plants merely showed mild male sterility, with a reduction of pollen germination, and AtSec24C knockdown plants showed an aberration in female gametogenesis (Tanaka et al., 2013). These results suggest that the functional diversification of plant COPII components occurred in the regulation of the plant early secretory pathway to maintain the dynamic identity of secretory organelles.

In recent studies of plant vesicular trafficking, the spatial relationship between the ERES and Golgi apparatus has been a matter of controversy because exit from the ER has been difficult to visualize, and interpretations of the same observations have not necessarily reached a consensus. To explain these contradictions, two typical models have been proposed by two groups about the organelle relationship (Dasilva et al., 2004; Yang et al., 2005). One model predicts that protein export from the ER occurs via the sequential recruitment of inner and outer COPII components to form transport intermediates at the mobile, Golgi-associated ERES. The other model predicts that the Golgi apparatus is not continually linked to a single ERES; instead, Golgi stacks associate intermittently and sometimes concurrently with several ERES as they are moving around in the cells. It was proposed that general differences in Golgi motility between plant leaves and suspension cells could be the reason for these discrepancies (Marti et al., 2010). More recently, to investigate plant ER import site (ERIS) where retrograde COPI vesicle fuse, fluorescence imaging experiments were performed and revealed that a Golgi-associated mobile domain of the ER, which is labeled by SYP72-YFP, play pivotal roles in COPI vesicle fusion and COPII vesicle budding as a common platform (Langhans et al., 2012; Lerich et al., 2012). Furthermore, COPI vesicle fusion with the ER is restricted to periods when Golgi stacks are stationary, but that when moving both COPII and COPI vesicles are tethered and collected in the ER-Golgi interface. These findings established a new model where the Golgi stack and an associated domain of the ER thereby constitute a mobile secretory and recycling unit (Langhans et al., 2012; Lerich et al., 2012). This is a characteristic feature in plant cells. Most recently, another imaging study using yeast system reported that direct contact of *cis*-Golgi with the ERES executes cargo capture and delivery from the ER (Kurokawa et al., 2014). The structural relationship between the mobile unit system in plants and the direct contact system in yeast remains to be clarified in future studies.

In higher plants, a functional differentiation has emerged in the mechanism of protein export from the ER (Gonzalez et al., 2005). In *Arabidopsis*, the PHOSPHATE TRANSPORTER1 (PHT1) gene family encodes phosphate (Pi) transporters that play a fundamental role in Pi acquisition and remobilization in plants. Mutation of PHOSPHATE TRANSPORTER TRAFFIC FACILITATOR1 (PHF1) caused impairment of Pi transport, resulting in the constitutive expression of many Pi starvation-induced genes and reduced Pi accumulation (Gonzalez et al., 2005). PHF1 encodes a plant-specific protein family conserved in *Arabidopsis*, rice and tomato. Their protein structures are related to the SEC12 proteins but lack most of the conserved domains of SEC12 proteins essential as guanine nucleotide exchange factors. *Arabidopsis* PHF1 was found to be localized to the ER, and its mutation caused the ER retention and reduced accumulation of the plasma membrane transporter PHT1;1. In contrast, both the plasma membrane localization and secretion of other proteins were not affected in this mutant. These results indicate that plants have evolved a novel mechanism that enables the cargo-specific protein export of Pi transporters from the ER.

### Plant Arf proteins and their interacting proteins

The fact that 12 Arf proteins exist in *Arabidopsis* indicates that functional specialization has substantially occurred in the plant Arf protein family (Table 2). In addition to this, when taken into consideration that the single Arf1 protein executes multiple functions, including roles in Golgi-to-ER retrograde traffic and post-Golgi traffic in yeast (Yahara et al., 2001), it is quite difficult to elucidate all the functions of plant Arf proteins. Therefore, limited information has accumulated for plant Arf proteins.

**Table 2:** ***Arabidopsis* Arf proteins and their interacting proteins**.

**Protein**	**Other name**	**AGI numbers**	**Function or putative function**
AtARFA1a	ARF1A/AtArf1	At1g23490	Small GTPase
AtARFA1b	ARF1A	At5g14670	Small GTPase
AtARFA1c	ARF1A/BEX1	At2g47170	Small GTPase
AtARFA1d	ARF1A	At1g70490	Small GTPase
AtARFA1e	ARF1A	At3g62290	Small GTPase
AtARFA1f	ARF1A	At1g10630	Small GTPase
AtARFB1a	ARF1B/ARFB	At2g15310	Small GTPase
AtARFB1b	ARF1B	At5g17060	Small GTPase
AtARFB1c	ARF1B	At3g03120	Small GTPase
AtARFC1	ARF1C	At3g22950	Small GTPase
AtARFD1a	ARF1D	At1g02440	Small GTPase
AtARFD1b	ARF1D	At1g02430	Small GTPase
Coatomer α		At1g62020	Coat protein for COPI vesicle
		At2g21390	
Coatomer β		At4g31480	Coat protein for COPI vesicle
		At4g31490	
Coatomer β′		At1g52360	Coat protein for COPI vesicle
		At3g15980	
		At1g79990	
Coatomer γ		At4g34450	Coat protein for COPI vesicle
Coatomer δ		At5g05010	Coat protein for COPI vesicle
Coatomer ε		At2g34840	Coat protein for COPI vesicle
		At1g30630	
Coatomer ζ		At1g60970	Coat protein for COPI vesicle
		At3g09800	
		At1g08520	

Thus far, the ARF1A subclass is the best-characterized group of the *Arabidopsis* Arf protein family (Robinson et al., 2007). One of the *Arabidopsis* Arf1A proteins can complement the lethality of the yeast *arf1 arf2* deletion mutant, and its GFP-fusion is localized to the Golgi apparatus in plant cells, as is its animal counterpart (Takeuchi et al., 2002). *In vitro* COPI-vesicle-generation experiments have demonstrated that Arf1A as well as γ-COP could be recruited from the cytosol onto mixed ER/Golgi membranes in cauliflower. The presence of plant COPI vesicles was confirmed by *in vitro* vesicle budding assays coupled with immunogold negative staining (Pimpl et al., 2000). Molecular approaches utilizing the transient expression of GDP- or GTP-locked *Arabidopsis* Arf1A mutants caused an abrogation of ER-to-Golgi transport and a redistribution of GFP- or YFP-tagged Golgi membrane marker proteins into the ER in plant cells (Lee et al., 2002; Takeuchi et al., 2002; Stefano et al., 2006). These results suggests that plant Arf1A proteins execute highly conserved functions in the formation of COPI vesicles at the Golgi apparatus by recruiting COPI coat complexes to the membranes (Letourneur et al., 1994; Pimpl et al., 2000; Robinson et al., 2007).

In addition to the functional Golgi localization of Arf1A proteins, there is strong evidence that Arf1A localizes to the trans-Golgi network (TGN) and FM4-64 positive compartments (endosomes). A protoplast-based transient expression study showed that Arf1A proteins are required for the post-Golgi sorting of soluble vacuolar proteins, implying that Arf1A is involved in clathrin-coated vesicle formation at the TGN (Pimpl et al., 2003). Visualization of the TGN localization of Arf1A was provided by immunofluorescence studies using transgenic *Arabidopsis* lines expressing SYP61–CFP and VHA-a1-GFP as TGN markers (Paciorek et al., 2005; Tanaka et al., 2009). Other expression studies using Arf1A-GFP and immunogold electron microscopy demonstrated that Arf1A colocalizes in FM4-64 positive compartments that are distinct from the Golgi apparatus (Xu and Scheres, 2005; Stierhof and El Kasmi, 2010). In contrast, a recent study of plant-powdery mildew interactions reported that barley (*Hordeum vulgare*) ARFA1b/1c is localized to multivesicular bodies (MVBs) and required for callose deposition in papillae, leading to penetration resistance against pathogenic fungi (Bohlenius et al., 2010). However, because supporting evidence for this MVB localization is insufficient, it remains unclear whether Arf1A is localized to MVB compartments (Robinson et al., 2011). Therefore, these results indicate that the single Arf1A protein exerts multiple functions in Golgi-to-ER retrograde traffic, post-Golgi traffic and endocytic traffic in plant endomembrane trafficking.

Although Arf1A proteins have been studied primarily at the single-cell level, the developmental functions of the ARF1A subclass of the *Arabidopsis* Arf family are still unknown at the whole-plant or tissue level. This is because six virtually identical ARF1A genes were found to be ubiquitously expressed and single loss-of-function mutants in these genes revealed no obvious developmental phenotypes. To address the mechanism determining the apical-basal polarity of epidermal cells during plant developmental processes, dominant-negative Arf1A mutants were conditionally expressed by a heat-shock-inducible system in transgenic *Arabidopsis*. The expression of GDP- or GTP-locked Arf1A mutants caused the abolition of root hair outgrowth at the early stages of root epidermal cell differentiation (Xu and Scheres, 2005). However, unlike in GDP-locked Arf1A-mutant-expressing lines, proper root hair formation recovered abruptly in GTP-locked Arf1A-mutant-expressing lines within 2 days, indicating that the effects of the GTP-locked Arf1A mutant on root hair formation were reversible. This difference in the effects caused by the GTP- and GDP-locked mutants of Arf1A on epidermal cell polarity might suggest that they act on different target molecules, with distinct properties in protein level or stability. The heat-shock-inducible expression study also revealed that the plasma membrane localization of a GFP-tagged auxin transporter, PIN2-GFP, was slowly affected upon Arf1A manipulation when compared with that of Golgi and endocytic markers. These studies enabled the dissection of the Arf1A functions involved in local and specific aspects of cell polarity (Xu and Scheres, 2005).

Plant Arf proteins are key molecules in the regulation of vesicular trafficking in the multicellular development of plants and are postulated to control both household functions and plant-specific functions through interactions with their interacting proteins. Nonetheless, most of the molecular mechanisms of plant-specific functions are still being elucidated. Unlike Arf1A proteins, which are targeted to the Golgi and post-Golgi structures, one of the ARF1B subclass proteins, ARFB, is localized to the plasma membrane (PM) (Matheson et al., 2008). This PM localization is a similar feature with mammalian Class III Arf6 proteins, which specifically play crucial roles in endocytic transport with specific regulators, such as EFA6 and SMAP1, in mammalian systems (Macia et al., 2001; Tanabe et al., 2005). However, it is largely unknown whether ARFB is involved in the regulation of plant-specific functions, including endocytic transport, because information about ARFB is insufficient. The functions and localization of the plant Arf proteins belonging to other subclasses are largely unknown.

Recent studies have revealed that the functional differentiation of the regulator proteins for plant Arf proteins could contribute to the plant-specific functions of vesicular traffic (Table 3). The *Arabidopsis* Arf-GAP protein family consists of 15 members containing the conserved GAP domain, designated ARF-GAP domain (AGD) proteins (Vernoud et al., 2003). Each AGD protein localizes to its specific cellular membrane compartment. For instance, AGD7 localizes to the Golgi apparatus, where its overexpression was found to inhibit the Golgi localization of γ-COPs and to induce the relocation of Golgi membrane proteins into the ER in both protoplasts and transgenic plants (Min et al., 2007). Its closely related homologs, AGD8 and AGD9 also localize to the Golgi and are required for the maintenance of Golgi morphology (Min et al., 2013). Gene knockdown experiments by RNA interference revealed that low-level expression of AGD8 and AGD9 induced abnormal Golgi morphology, inhibition of protein trafficking, and arrest of plant growth and development. Conversely, high-level expression of AGD8 and AGD9 evoked Arf1A recruitment to the Golgi and suppressed Golgi disruption and vacuolar trafficking defects that were caused by overexpression of AGD7 (Min et al., 2007, 2013). Imaging studies using protoplasts showed that AGD7, AGD8 and AGD9 can recruit a GDP-locked Arf1A mutant (Arf1 T31N) from the cytosol to the Golgi. Thus, the Golgi-localized ARF-GAPs (AGD7, AGD8 and AGD9) fulfill redundant functions in Arf1A-mediated protein trafficking, which is essential for plant development and growth. Another AGD protein, AGD5, is localized to the TGN, where it co-localizes with Arf1A proteins, central GTPases that play essential roles in plant membrane trafficking at the Golgi and post-Golgi structures. The transient expression of a mutant AGD5 protein having impairment in ARF-GAP activity caused longer recruitment of Arf1A on the membranes, indicating that the GTP hydrolysis of Arf1A was impaired due to a defective GAP (Stefano et al., 2010). These results define a role of AGD5 for Arf1A as an ARF-GAP localizing at the TGN.

**Table 3:** **Regulators for *Arabidopsis* Arf proteins**.

**Protein**	**Other name**	**AGI numbers**	**Function or putative function**
AGD1		At5g61980	GTPase-activating protein (GAP) for Arf
AGD2		At1g60680	GTPase-activating protein (GAP) for Arf
AGD3	VAN3/SCARFACE/SFC	At4g13300	GTPase-activating protein (GAP) for Arf
AGD4		At1g10870	GTPase-activating protein (GAP) for Arf
AGD5	NEV/MTV4	At5g54310	GTPase-activating protein (GAP) for Arf
AGD6		At3g53710	GTPase-activating protein (GAP) for Arf
AGD7		At2g37550	GTPase-activating protein (GAP) for Arf
AGD8		At4g17890	GTPase-activating protein (GAP) for Arf
AGD9		At5g46750	GTPase-activating protein (GAP) for Arf
AGD10	RPA	At2g35210	GTPase-activating protein (GAP) for Arf
AGD11	CML3/CALMODULIN-LIKE3	At3g07490	GTPase-activating protein (GAP) for Arf
AGD12	ZAC	At4g21160	GTPase-activating protein (GAP) for Arf
AGD13		At4g05330	GTPase-activating protein (GAP) for Arf
AGD14	ZIGA4	At1g08680	GTPase-activating protein (GAP) for Arf
AGD15		At3g17660	GTPase-activating protein (GAP) for Arf
Sec7-type		At4g35380	Guanine nucleotide exchange factor (GEF) for Arf
ARF-GEF		At4g38200	
		At1g01960	
		At3g60860	
		At3g43300	
GNOM-type		At1g13980 (GNOM)	Guanine nucleotide
ARF-GEF		At5g39500 (GNL1)	exchange factor
		At5g19610 (GNL2)	(GEF) for Arf

Several AGD proteins are specifically expressed in particular plant tissues and play essential roles in tissue formation. AGD12/ZAC has a novel domain structure in which the N-terminal ARF-GAP domain containing a zinc finger domain and a C-terminal C2 domain are separated by a region without homology to other known proteins (Jensen et al., 2000). Expression analyses using a Zac promoter/beta-glucuronidase reporter revealed the highest expression levels in flowering tissues, rosettes and roots. The ZAC protein was mainly associated with membranes that were co-fractionated with Golgi and plasma membrane marker proteins. Recombinant ZAC was found to possess GTPase-activating activity on *Arabidopsis* Arf1A proteins, and the ZAC N-terminal region has a significant binding activity for phosphatidylinositol 3-monophosphate. These data indicate a role for ZAC in the regulation of ARF-mediated vesicular traffic. Another type of AGD protein, AGD1, is an ARF-GAP containing a phosphoinositide binding pleckstrin homology (PH) domain protein. *agd1* mutants have root hairs that exhibit wavy growth and have two tips that originate from a single initiation point. These root hair defects were associated with the bundling of microtubules and filamentous actin that extended to the root hair apex. Characterization of the *agd1* mutant provided evidence that AGD1 plays crucial roles in root hair development through the cross-talk among phosphoinositides, cytoskeleton and other signals mediated by other GTPases (Yoo et al., 2012; Yoo and Blancaflor, 2013).

A different type of AGD protein, VAN3/AGD3, contains four domains: a BAR (BIN/amphiphysin/RVS) domain, a PH domain, an ARF-GAP domain and an ankyrin (ANK)-repeat domain. VAN3 plays a pivotal role in plant venation continuity, and the recombinant protein showed GTPase-activating activity on Arf1A and a specific affinity for phosphatidylinositol. VAN3 localizes at the plasma membranes as well as in intracellular structures, including the TGN (Koizumi et al., 2005; Naramoto et al., 2009, 2010). Single-molecule fluorescence imaging showed that VAN3 localizes to discrete foci at the plasma membrane that are associated with the endocytic vesicle coat protein clathrin. Imaging studies using transgenic plants revealed that VAN3 activities are required for the endocytosis and internalization of plasma membrane proteins, including PIN-type auxin transporters. The functions and localization of other AGD proteins remain to be elucidated.

The *Arabidopsis* ARF-GEF protein family consists of 8 members containing the conserved Sec7 domain, and they are classified into two groups: Sec7-type and GNOM-type groups (Robinson et al., 2007) (Table 3). When compared with animal ARF-GEFs, all the *Arabidopsis* ARF-GEFs belong to the GBF/BIG family of animal ARF-GEFs, whereas no ARNO family exists in *Arabidopsis*. This is a plant-specific feature of ARF-GEF divergence. *Arabidopsis gnom/emb30* mutants were isolated as embryonic-lethal mutants having impairments in the first zygotic cell division and apical-basal pattern formation during early developmental processes (Shevell et al., 1994). These mutant phenotypes are very similar to the inhibition of the polarized transport of the plant hormone auxin. The GNOM/EMB30 gene encodes an ARF-GEF protein that localizes to endosomes and contributes to their structural integrity. This protein is a brefeldin A (BFA)-sensitive ARF-GEF that is required for the proper polar localization of an auxin transporter, PIN1. A molecular approach utilizing an engineered BFA-resistant version of GNOM demonstrated that GNOM is specifically required for the recycling of auxin transport components from endosomes. In contrast, the *Arabidopsis* GNOM-LIKE1 (GNL1) protein is a BFA-resistant ARF-GEF that localizes to the Golgi but is also required for selective internalization from the plasma membrane in the presence of BFA. This is consistent with experimental results that the internalization of an auxin efflux carrier, PIN2, was selectively inhibited in BFA-treated *gnl1* roots. Taken together, these results suggest that both GNOM and GNL1 proteins are involved in the selective endocytosis of auxin transport components, indicating that the evolution of endocytic trafficking in plants is correlated with the neofunctionalization of GNOM-type ARF-GEFs (Geldner et al., 2003; Teh and Moore, 2007).

GNL1 and GNOM are multi-functional proteins and execute multiple roles in protein trafficking. Molecular-genetic experiments based on introduction of an engineered BFA-sensitive GNL1 into a *gnl*1 knockout background revealed that both a block in ER-Golgi traffic and a release of γ-COP into the cytosol were induced by addition of BFA (Richter et al., 2007). These results suggest that GNL1 is specifically required for retrograde transport from the Golgi to the ER and maintenance of Golgi integrity. GNL1 is one of the major regulators of ER-Golgi traffic but one or more BFA-sensitive ARF-GEFs are needed for its regulation because a *gnl*1 knockout mutant exhibits no defects in ER-Golgi traffic and Golgi integrity (Richter et al., 2007). The most recent study indicates that GNOM primarily localizes to the Golgi apparatus and that GNOM and GNL1 are colocalized at distinct subdomains on Golgi cisternae (Naramoto et al., 2014). Short-term BFA treatment stabilizes GNOM at the Golgi, whereas prolonged exposures results in GNOM translocation to the TGN/endosomes. These data are supported by the fact that GNOM can partially replace the function of GNL1 in the Golgi to ER retrograde transport, implying that GNOM might be a minor regulator of ER-Golgi traffic (Richter et al., 2007).

As for cargo specificity of endocytotic traffic, *gnom* and *gnl*1 mutants showed complicated phenotypes. In *gnom* mutant lines expressing BFA-resistant GNOM, a cytokinesis-specific syntaxin KNOLLE was still aggregated into large BFA bodies in the presence of BFA, suggesting that KNOLLE traffic in cytokinetic cells is dependent on other BFA-sensitive ARF-GEFs (Geldner et al., 2003). In contrast, PIN2 and PM-ATPase showed a partial BFA-resistance in the same lines, indicating that the recycling rates or transport routes of these molecules might be different between individual cells and roots (Geldner et al., 2003). In the case of *gnl1* mutants, internalization of other molecules except PIN2 can occur normally in the absence of GNL1 function. A plasma-membrane marker PMA4-GFP and a lipophilic dye FM4-64 are accumulated in the BFA bodies of BFA-treated wild-type and *gnl1-2* roots with similar efficiency (Teh and Moore, 2007). These results suggest that GNL1 is not a major regulator of internalization from the plasma membrane but one or more BFA-sensitive ARF-GEFs play crucial parts in its regulation. Thus, GNL1 and GNOM execute distinct but overlapping functions in plant vesicular traffic. However, the molecular mechanisms underlying cargo specificity of the GNL1/GNOM-mediated endocytosis remain poorly elucidated. Further functional analyses coupled with molecular characterizations of other ARF-GEF proteins are necessary to elucidate fundamental aspects of ARF-GEF-mediated traffic.

Another GNOM-type ARF-GEF, GNOM-LIKE2 (GNL2), is highly expressed in pollen grains and pollen tubes. In addition, pollen germination defects were observed in the corresponding *Arabidopsis* mutants, *gnom-like 2-1* (*gnl2-1*) and *gnl2-2*. These results suggest that GNL2-related traffic plays an important role in pollen germination (Jia et al., 2009). Thus, ARF-GEF-mediated vesicular trafficking is tightly correlated with plant developmental processes, including early embryonic development and sexual plant reproduction (Jia et al., 2009; Du et al., 2013).

## Concluding remarks

Extensive studies by means of genetic and biochemical approaches have given rise to much progress in the understanding of the molecular mechanisms by which Sar/Arf GTPases drive vesicle formation on membrane surfaces. As discussed above, many questions remain to be answered. In particular, limited information is available on the physiological functions of Sar/Arf in plants. These GTPases achieve both housekeeping conserved functions and plant-specific functions in vesicular trafficking. To date, many unique functional differentiations have been described regarding such processes as Golgi organization, endocytic transport and cell polarity. However, further elucidation of these phenomena is required for a better understanding of multicellular development in higher plants.

### Conflict of interest statement

The authors declare that the research was conducted in the absence of any commercial or financial relationships that could be construed as a potential conflict of interest.
